# Recombinant growth hormone therapy in a girl with costello syndrome: a 4-year observation

**DOI:** 10.1186/s13052-015-0209-4

**Published:** 2016-01-26

**Authors:** Ewa Blachowska, Elżbieta Petriczko, Anita Horodnicka-Józwa, Agata Skórka, Magdalena Pelc, Małgorzata Krajewska-Walasek, Mieczysław Walczak

**Affiliations:** Department of Pediatrics, Endocrinology, Diabetology, Metabolic Diseases and Cardiology of the Developmental Age, Pomeranian Medical University in Szczecin, Ulica Unii Lubelskiej 1, 71-252 Szczecin, Poland; Department of Medical Genetics, The Children’s Memorial Health Institute, Warsaw, Poland; Department of Pediatrics, Medical University of Warsaw, Warsaw, Poland

**Keywords:** Costello syndrome, Growth hormone deficiency, Hypertrophic cardiomyopathy, Malignancy, *HRAS* gene

## Abstract

**Background:**

Costello syndrome is a rare syndrome of multiple congenital anomalies. The typical clinical traits include dysmorphic craniofacial features, skin hyperpigmentation and excess, feeding difficulties leading to severe postnatal growth retardation, short stature, joint hypermobility, and delayed psychomotor development. Additionally, Costello syndrome may present with an increased incidence of congenital heart disease, hypertrophic cardiomyopathy, and increased risk of both benign and malignant tumors. Furthermore, cases of patients with endocrine disorders such as adrenal insufficiency and endogenous growth hormone deficiency have also been documented.

**Case presentation:**

We present a patient with Costello syndrome who has been successfully treated with recombinant human growth hormone (rhGH) for almost 4 years.

**Conclusions:**

The possibility of growth hormone (GH) treatment can be considered in cases of documented GH deficiency in patients with Costello syndrome, but only under close oncologic and cardiologic supervision.

## Background

Costello syndrome (CS, OMIM #218040) is caused by heterozygous germline mutations in the proto-oncogene HRAS that cause dysfunction of the Ras-MAPK signaling pathway. To date, 15 mutations in HRAS have been identified. The birth prevalence of this disease is estimated at 1:1,230,000 to 1:300,000 [1–4]. Clinically, CS is characterized by polyhydramnios, high birth weight, postnatal growth retardation, relative macrocephaly, coarse facial features, loose skin, especially of the hands and feet, hyperpigmentation, hypertrophic cardiomyopathy, atrial arrhythmias, papillomata, developmental delay or mental retardation, and predisposition to malignancies. In the newborn and neonatal periods, the presence of suggestive facies and severe feeding difficulties leading to failure to thrive and hypoglycemia help make the correct diagnosis [5–7]. Furthermore, cases of Costello syndrome patients with endocrine disorders such as adrenal insufficiency and endogenous growth hormone deficiency have also been documented in the literature [8–11]. Due to the complex nature of the discussed syndrome, patients require multidisciplinary care (provided by cardiologists, speech therapists, gastroenterologists, orthopedic surgeons) along with early stimulation and developmental support. Because of the short stature and growth hormone deficiency in this condition, growth hormone therapy is often considered. For decades there has been great debate on the anticipated risk of carcinogenesis and cardiomyopathy weighted against the potential benefits resulting from recombinant human growth hormone (rhGH) therapy. To date, there are no conclusive data showing a negative role of rhGH therapy in the development of these diseases.

Here we report a six-year-old patient with Costello syndrome, who has been successfully treated with rhGH for 42 months.

## Case presentation

AM, was born at 40 weeks gestation by caesarean section (due to large fetal mass) as the first baby of healthy and nonconsanguineous parents with a birth weight of 4270 g (>97 c), length of 55 cm (50 c) and head circumference of 38 cm (>97c). Her Apgar score was 7/7/8 points. The pregnancy was complicated by polyhydramnios and impaired glucose tolerance in the mother.

In the neonatal period, generalized edema, hypoglycemia, and hypocalcaemia were observed. Transthoracic echocardiography revealed thickened muscles of both ventricles and septum, although follow-up cardiac examinations revealed no evidence of cardiomyopathy.

During the first two years of life the child was repeatedly hospitalized due to a number of problems (the clinical symptoms observed in the patient are presented in Table 1). At the age of 8 weeks the girl was evaluated due to failure to thrive. Physical examination revealed: poorly developed subcutaneous tissue with excess skin (this was attributed to resolution of edema), reduced muscle tone, and enlarged liver. Laboratory tests did not find evidence of hypothyroidism or inborn errors of metabolism. Ultrasonography followed by gastric scintiscan and upper GI series revealed hiatal hernia and gastric endoscopy, the latter being the base for a diagnosis of gastroesophageal reflux. Additionally, the 24-h esophageal pH study was conducted, to further help with diagnosis, yielding inconsistent results that were borderline normal. At the age of four months the patient manifested psychomotor delay, joint laxity, variable muscle tone, and hyperactive knee reflexes. Follow-up cardiac examinations revealed no signs of cardiomyopathy, but complex premature ventricular and supraventricular contractions and sinus arrest were present. She required treatment with amiodarone, which was withdrawn after one month. No cardiac arrhythmias were shown in subsequent 24-h-Holter monitoring.

**Table 1:** Symptoms in consecutive months of life

Child’s age	Clinical symptoms														
	Lack of appetite	Vomiting, spitting up	Dysphagia	Esophagitis	Underweight	Hepatomegaly	Neurological abnormalities	Delayed speech development	Loose skin	Coarse facial features	Arrhythmias	Hypertrophic cardiomyopathy	Excessive joint laxity	Achilles tendon contractures	Short stature
1 month									^a^			+^b^			
2−3 months	+	+	+	+		+			+						
4−6 months	+	+	+	+	+		+^d^		+		+^c^		+		
7−9 months	+	+	+	+	+		+^d^	+	+				+		
11−24 months	+	+	+	+	+			+	+	+			+		+
24−36 months					+			+	+	+			+		+
>3 years of life								+	+	+			+	+	+

Key

^a^Edema

^b^Suspicion, later studies did not confirm hypertrophic cardiomyopathy

^c^Transient

^d^Reduced muscle tone, periodic spasticity of the lower limbs

The treatment of gastroesophageal reflux, which involved proton pump inhibitors and prokinetic agents, was ineffective. During eight months, her weight gain had been only 1000 g. At the age of nine months the patient weighed 5300 g (SD: −4.92). Due to persistent feeding difficulties she was referred for surgical treatment and Nissen fundoplication was performed at the age of 11 months. The improvement after abovementioned procedure was very brief, with feeding difficulties quickly reoccurring. Due to lack of improvement after pharmacological treatment, and persistent signs of gastroesophageal reflux in performed endoscopy our patient was qualified for second operation. Finally the decision about surgical treatment was abandoned due to slow spontaneous improvement in feeding and apetite after the patient was 3 years old.

**Fig. 1:**
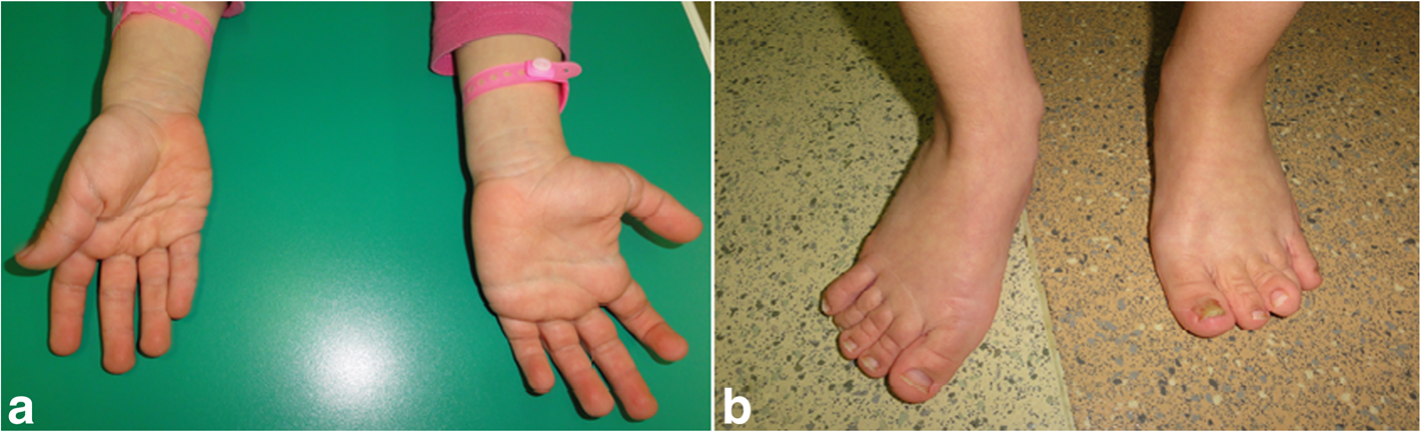
The presented 6-year-old girl with Costello syndrome after 30 months of treatment with rhGH. The pictures show typical phenotypic features of the syndrome: loose skin with deep palmar creases (**a**); abnormal feet position as a result of excessive joint laxity (**b**)

At the age of 2 years and 9 months Costello syndrome was suspected on the basis of phenotypic traits (large mouth, wide lips, short nasal bridge, loose skin deeply fissured on the palms and soles of the feet, joint laxity, and abnormal feet position, Fig. 1) and case history. The diagnosis was confirmed by molecular tests, which identified c.35G > C (p.G12A) substitution in the HRAS gene, the second most common mutation responsible for Costello syndrome. Molecular studies in both parents revealed that this mutation occurred de novo [3, 4].

The investigation of delayed growth was performed at the age of 3 years and 2 months. Physical examination noted significantly short stature, i.e., a height of 84 cm (SD: −4.33), and underweight, 10.7 kg (SD: −2.86). Routine laboratory tests did not show any abnormalities. Electrocardiographic examination and abdominal ultrasound were normal. Hormone analysis revealed normal thyroid function, normal daily cortisol profile and rhythm. Reduced levels of *insulin*-like growth factor 1 in relation to chronological age were detected (26,3 ng/ml, normal level range at this age 49–289 ng/ml). Bone age was found to be consistent with the chronological age. Growth hormone levels did not reach normal values in the nocturnal secretion test or in L-Dopa and clonidine stimulation tests (maximum secretion: 8 ng/ml), see Table 2 for details. Based on the above findings and clinical observations, partial endogenous growth hormone deficiency was established.

**Table 2:** Growth hormone secretion in nocturnal and stimulation tests

	0’	30’	60’	90’	120’	150’
Nocturnal GH excretion [ng/ml]	4,4	2,1	0,8	1,7	2,0	1,9
GH after L-dopa [ng/ml]	0,94	0,99	1,1	1,3	1,2	0,88
GH after clonidine [ng/ml]	0,91	0,96	5,2	8,0	4,0	2,5

At the age of 3 years and 9 months the girl was qualified for growth hormone therapy, following careful considerations of the risks and benefits and discussion of the issue with the child’s parents.

Growth hormone was administered by subcutaneous injection once daily, in the evening, at a dose of 0.031 mg/kg/day. Initially, the check-up visits in the Department were held every three months, and then every six months. The following issues were evaluated: response to treatment, tolerability, and laboratory test results, with particular emphasis on carbohydrate metabolism and bone age progression (Table 3).

**Table 3:** Selected auxological parameters of the patient during rhGH treatment

Age [years and months]	Body height [cm]	Body height SDS	Growth rate [cm/year]	Body weight [kg]	Body weight SDS	BMI [kg/m^2^]	BMI SDS	Bone age [years and months]
3 years and 2 months	84	−4.94	2.10	10.7	−2.86	15.2	−0.504	
3 years 9 months (start of rhGH treatment)	84.7	−4.92	0.80	12.1	−2.60	16.9	1.005	3 years
4 years	88.9	−3.48	16.80	12.1	−2.58	15.3	0.02	
4 years 5 months	92	−3.29	7.44	13.3	−2.41	15.7	0.496	
4 years 8 months	93.3	−3.31	7.40	14	−2.28	16.1	0.508	4 years 2 months
5 years 2 months	97.8	−2.84	5.20	15.4	−1.80	16.1	0.507	
5 years 6 months	103	−2.02	9.00	16	−1.77	15.1	0.475	5 years – 5 years 9 months
6 years 1 month	107.5	−1.67	13.00	19	−0.92	16.4	0.511	
6 years 8 months	109	−1.78	2.57 ^a^	18.4	−1.46	15.5	0.480	7 years 10 months
7 years 2 months	115	−1.61	12.00	20.3	−1.28	15.3	0.466	

^a^Interruptions in rhGH administration caused by orthopedic treatment

**Fig. 2:**
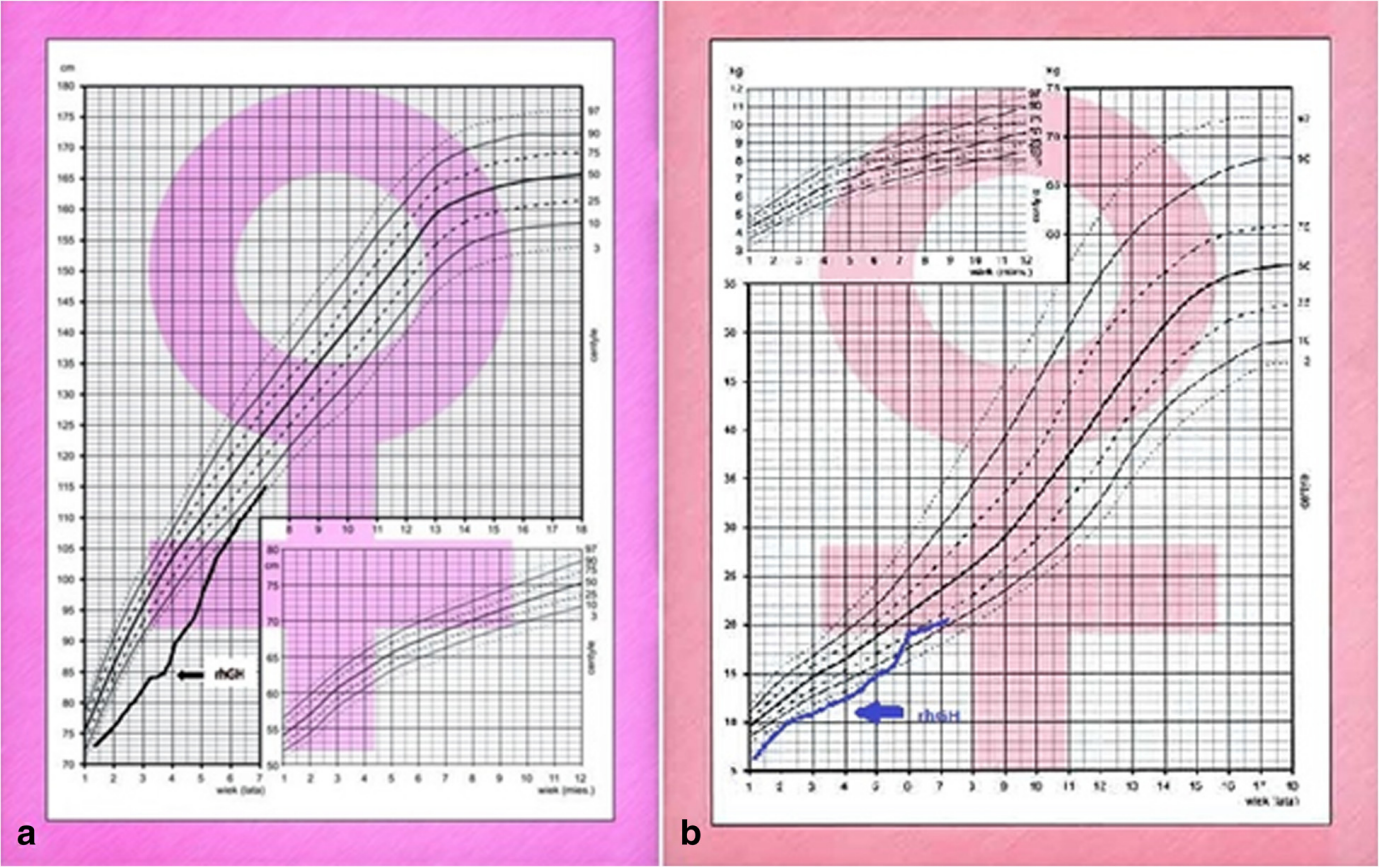
The course of growth (**a**) and weight gain (**b**) in the patient before and during rhGH treatment

During the initial check-ups, significant acceleration of growth was recorded. Throughout the first year of treatment, her growth rate was 9.49 cm/year, and in the next two years it was 12.36 cm/year and 7.71 cm/year, respectively, while during the pretreatment period her average growth rate was 4.28 cm/year. The curves of the patient's body weight and height gain before and during rhGH treatment are shown in Fig. 2a and b.

During the treatment, progression of the abnormalities within the ligaments of the ankles and hip joints (right club foot, left plano-valgus foot, shortening of the Achilles tendon, dislocation of the right hip) was observed. At the age of 5 years and 6 months, surgical correction of both feet was necessary due to further progression of mobility impairment, and plasty of the hip joint was performed in the following year. At present, the patient is recovering after the last intervention.

Currently, after 42 months of rhGH treatment, the girl has grown 30.3 cm, reaching a body height above the 3^rd^ percentile (115 cm; SD: −1.61), her weight gain has been 10.4 kg. Her current weight is 20.3 kg (10–25 percentile; SD: −1.28). So far, no serious adverse events have been observed. The patient tolerates daily injections of growth hormone well, she receives comprehensive speech therapy and rehabilitation, and attends kindergarten. Her parents have observed a significant improvement in her psychomotor development, especially with respect to speech.

## Discussion

Costello syndrome was first described in 1977 by Jack Costello, a New Zealand pediatrician, in two unrelated children who presented similar phenotypic characteristics. The molecular etiology of this syndrome (germline mutations in the HRAS gene) was identified in 2005 [2]. Among many dysfunctions associated with this condition, impaired growth is one of the most challenging clinical problems [8, 12, 13]. The average final height in Costello syndrome patients, without growth-stimulating therapy, reaches about 138 cm (118–148 cm) [7]. The pathogenesis of short stature associated with this condition has been studied in recent decades. The first cases diagnosed with partial/total endogenous growth hormone deficiency and treated with rhGH were described in the 1990s [9–11]. To date, there are few studies in the scientific literature that assess the safety and efficacy of rhGH therapy in individual patients [14, 15]. As the syndrome is extremely rare, evaluating a larger group of patients iseems to be a very difficult task. Increased incidence of cancer and increased risk of development of hypertrophic cardiomyopathy are serious clinical problems that should be taken into account when considering treatment with rhGH [1, 7, 8, 15]. Gripp et al. [5] estimated that the risk of developing tumors in patients with Costello syndrome reaches 17 %, most of which are rhabdomyosarcoma, neuroblastoma, papilloma, and bladder cancer. Interestingly, the p.G12A mutation detected in our patient is the main mutation associated with the higher risk of cancer in CS, which correlates with an up to 57 % risk in affected patients (2, 3). However, no occurrence of malignancy has been noted in the patient so far. Growth hormones promote cell divisions, which may generate a potential hazard. However, existing research in this area has given inconclusive results [14, 15]. Stein et al. [13] presented long-term observations involving three patients with endogenous growth hormone deficiency successfully treated with rhGH with no severe adverse events. On the other hand, cases of patients with unsatisfactory response to rhGH therapy [9] and complications during the treatment have also been documented. There are reports describing a case of a 16-year-old girl diagnosed with bladder cancer after 7 years of rhGH therapy [16], a 26-month-old boy diagnosed with rhabdomyosarcoma after one year of treatment [14], and progression of hypertrophic cardiomyopathy in a 6-year-old boy treated with rhGH [17]. However, in all of these cases it is difficult to assess whether the administration of growth hormone actually influenced the development of these diseases and to what extent.

During 3.5 years of follow-up, we have observed accelerated growth in our rhGH-treated patient, and no serious health complications have yet occurred. In infancy the girl was diagnosed with supraventricular arrhythmias and hypertrophic cardiomyopathy, which was not confirmed in subsequent echocardiography. However, because of the risk of recurrence she remains under the systematic control of a cardiologist. The observed motor-system problems, resulting from the excessive ligamentous laxity associated with Costello syndrome, were already present before the initiation of growth hormone therapy. It seems that their progression was associated with significant growth acceleration resulting from GH supply, but the impact of rhGH therapy alone is difficult to assess.

## Conclusions

The available data, based on a small number of reported cases of Costello syndrome treated with recombinant growth hormone, do not allow drawing general conclusions. The very good response to the treatment, demonstrated in our case, improved her body height prognosis and eliminated the disability related to extreme short stature. The 3.5-year observation revealed no serious side effects. However, reports of the otherauthors regarding the development and progression of cancer and hypertrophic cardiomyopathy in children with Costello syndrome treated with rhGH indicate that these patients require special oncologic and cardiologic supervision.

## Consent

Written informed consent was obtained from the patient for publication of this Case report and any accompanying images. A copy of the written consent is available for review by the Editor-in-Chief of this journal.

## Abbreviations

GH: Growth hormone
HRAS: Harvey rat sarcoma viral oncogene homolog
MAPK: Mitogen-activated protein kinases
rhGH: Recombinant human growth hormone
SD: Standard deviation
GI: Gastrointestinal
